# Evaluating synthetic odours and trap designs for monitoring *Anopheles farauti* in Queensland, Australia

**DOI:** 10.1186/s12936-019-2923-7

**Published:** 2019-09-02

**Authors:** Bram van de Straat, Alexandra Hiscox, Willem Takken, Thomas R. Burkot

**Affiliations:** 1 Laboratory of Entomology, Wageningen University and Research, Wageningen, The Netherlands; 2 ARCTEC, London School of Hygiene and Tropical Medicine, London, UK; 3 Australian Institute of Tropical Health and Medicine, James Cook University, Cairns, Australia

**Keywords:** *Anopheles farauti*, Vector surveillance, Synthetic odours, Fan-powered trapping

## Abstract

**Background:**

Monitoring of malaria vectors is important for designing and maintaining effective control interventions as changes in vector-feeding habits can threaten the efficacy of interventions. At present, human landing catches remain the most common method for monitoring malaria vectors of the *Anopheles punctulatus* complex, including the *Anopheles farauti* group. The aims of this study were to evaluate the efficacy of different lures and fan-powered traps, including an odour blend that has been demonstrated to be attractive to African anophelines, in Queensland, Australia.

**Methods:**

To evaluate the performance of different lures in trapping *An. farauti* in the field, four Suna traps were baited with either: CO_2_-alone, a synthetic lure (MB5 or BG-Lure) plus CO_2_, or a human odour plus CO_2_ and set in the field in Cairns, eastern Australia. A second study evaluated the performance of four traps: a Passive Box trap, BG-Suna trap, BG-Sentinel 2 trap, and BG-Bowl trap, for their ability to trap *An. farauti* using the best lure from the first experiment. In both experiments, treatments were rotated according to a Latin square design over 16 nights. Trapped mosquitoes were identified on the basis of their morphological features.

**Results:**

BG-Suna traps baited with CO_2_ alone, a BG-Lure plus CO_2_ or a natural human odour plus CO_2_ captured comparable numbers of *An. farauti*. However, the number of *An. farauti* sensu lato captured when the MB5 lure was used with CO_2_ was three times lower than when the other odour lures were used. The BG-Sentinel 2 trap, BG-Suna trap and BG-Bowl trap all captured high numbers of *An. farauti*, when baited with CO_2_ and a BG-Lure. The morphological condition of captured mosquitoes was affected by mechanical damage caused by all fan-powered traps but it was still possible to identify the specimens.

**Conclusions:**

The BG-Sentinel 2 trap, BG-Suna trap and the BG-Bowl trap captured high numbers of *An. farauti* in the field, when equipped with CO_2_ and an odour lure (either the BG-Lure or a natural odour). The most important attractant was CO_2_. This study shows that fan-powered traps, baited with CO_2_ plus an appropriate odour lure, can be a promising addition to current vector monitoring methods in the Southwest Pacific.

## Background

Vector surveillance is an important component of the endeavour to eliminate malaria. An extensive and reliable vector surveillance network requires efficient, standardized methods for sampling adult vector populations, for monitoring of behavioural and physiological resistance to insecticides, to characterize biting behaviour, and to define the receptivity of different regions to local transmission of malaria.

Malaria control has recently stalled, with increases in malaria seen globally, including the Southwest Pacific [1]. There is a need for more effective monitoring of malaria vectors for changes in biting habits that can threaten the effectiveness of vector control strategies. The main vector species in the region belong to the *Anopheles punctulatus* complex, including *Anopheles farauti* sensu stricto (s.s.), which is the dominant vector in coastal areas from eastern Indonesia to Vanuatu [2, 3]. In the 1960s and 1970s, in response to indoor residual insecticide spraying (IRS) with DDT, *An. farauti* shifted its biting behaviour from all-night biting both indoors and outdoors to earlier evening and more outdoor biting [4–6]. Despite these behavioural adaptations, indoor interventions such as long-lasting insecticidal bed nets (LLINs) and IRS contribute significantly to malaria control in the Southwest Pacific because *An. farauti* is a species that typically feeds frequently and will enter houses late at night to blood feed [7] and will be likely to encounter LLINs or IRS.

Monitoring vector populations to determine endophagy, peak biting time, seasonality, and sporozoite infections rates requires a standardized, effective collection method. In the Southwest Pacific, the human landing catch (HLC) is still the ‘gold standard’, the most efficient (and often the only) surveillance method for collecting blood-seeking *An. farauti* sensu lato (s.l.). During HLCs, mosquitoes landing on a trained collector are captured as they begin to probe. The HLC targets anthropophagic anophelines, but is expensive, requiring collectors to work under close supervision with a risk of exposure to infectious mosquito bites [8]. Although mosquito collectors have been shown to not be at an increased risk of malaria (when provided with prophylaxis) [9], there is a possibility of exposure to arboviruses by biting culicines. To overcome these disadvantages, many mechanical trapping methods have been developed to replace the HLC. The Centers for Disease Control (CDC) Light Trap attracts mosquitoes with either an incandescent or ultraviolet light bulb and CO_2_ [10]. Where a source of CO_2_ is not available, the CDC light trap can be placed close to a (human) host, protected by a bed net, who provides CO_2_ and odorant lures [10]. Traps with an odour lure and CO_2_ that mimic a human host have been developed with an ultimate aim of replacing the HLC with a safer, cheaper, more standardized approach [11–14].

In these odour-baited traps, volatiles from the odour lure within the trap are emitted and spread by a constant airflow generated by a fan, while attracted mosquitoes are sucked into the trap. This counter-current concept has been applied in several traps, including the Mosquito Magnet traps (American Biophysics Corporation, USA) and the BG-Sentinel trap (Biogents AG, Germany). The inclusion of a synthetic odour lure would remove the need to place a trap close to a human host, thus enabling the trap to be used outdoors as well as indoors.

This first part of this study was conducted to evaluate the attraction of *An. farauti* to two synthetic lures, previously shown to be effective in attracting anthropophagic African anophelines, and a natural human odour sample. The most effective lure from the first part of the study was subsequently used to bait three different fan-powered traps and a passive trap to determine which trap was most effective in capturing *An. farauti* in tropical North Queensland, Australia.

## Methods

All field experiments were conducted in a rural area 15 km north of Cairns, Queensland, Australia, in June and July 2017 (16.8221°S, 145.6972°E). The site is situated in a swamp forest dominated by paperback (*Melaleuca*) trees, with human activity (suburbs, industrial areas, sugarcane farms) within a 1-km radius [15]. The climate is tropical, with hot, humid summers (November–March) and milder, dry winters (April–October). The min/max temperatures range from 23 °C/31 °C in summer to 18 °C/26 °C in winter, with an annual average rainfall of approximately 2000 mm. The prevailing wind direction in summer is north-eastern, dictated by the monsoon, and is dominated by the south-eastern trade winds during winter (data obtained from the Bureau of Meteorology, Australia).

### Lure comparison study

The attractiveness of four different lures was studied in June 2017. Four BG-Suna traps were baited with CO_2_ released at 250 ml/h plus either the BG-Lure in granular cartridge form (Biogents AG, Germany), the MB5 blend [16], an odour sample collected directly from a natural host on socks, or CO_2_ alone, at the same release rate (the control). CO_2_ was provided from a compressed gas tank, whereas the synthetic odour lures were released from either a cartridge (BG-Lure) or a sachet containing odorous crystals. The natural host odour was provided by a sock (95% cotton, 5% elastane), worn for 12 h by a human volunteer before the start of the experiment. To prevent possible alterations in the composition of odour compounds, a single sock was used during this experiment. All odour lures were wrapped in aluminium foil and stored at − 30 °C when not being used. To prevent contamination of the traps with multiple odours, each odour lure was assigned to a single trap.

The experimental design was a 4-by-4 Latin square trial, repeated four times for a total of 16 repetitions. To minimize the effects of the environment, four outdoor sampling locations were chosen which showed similar low ground vegetation patterns as well as bushes and trees. Traps were suspended from tree branches using rope with the inlet at 50 cm above ground level. Traps were separated by approximately 50 m to minimize competition from other traps that might have an effect on the number of mosquitoes caught. Mosquitoes were sampled between 16.00 and 08.00 h, thus covering the entire timespan of *An. farauti* biting activity. Each morning, traps were removed from the sampling sites and transferred to the laboratory, where mosquitoes were frozen at − 30 °C prior to morphological identification [17].

### Trap comparison study

**Fig. 1:**
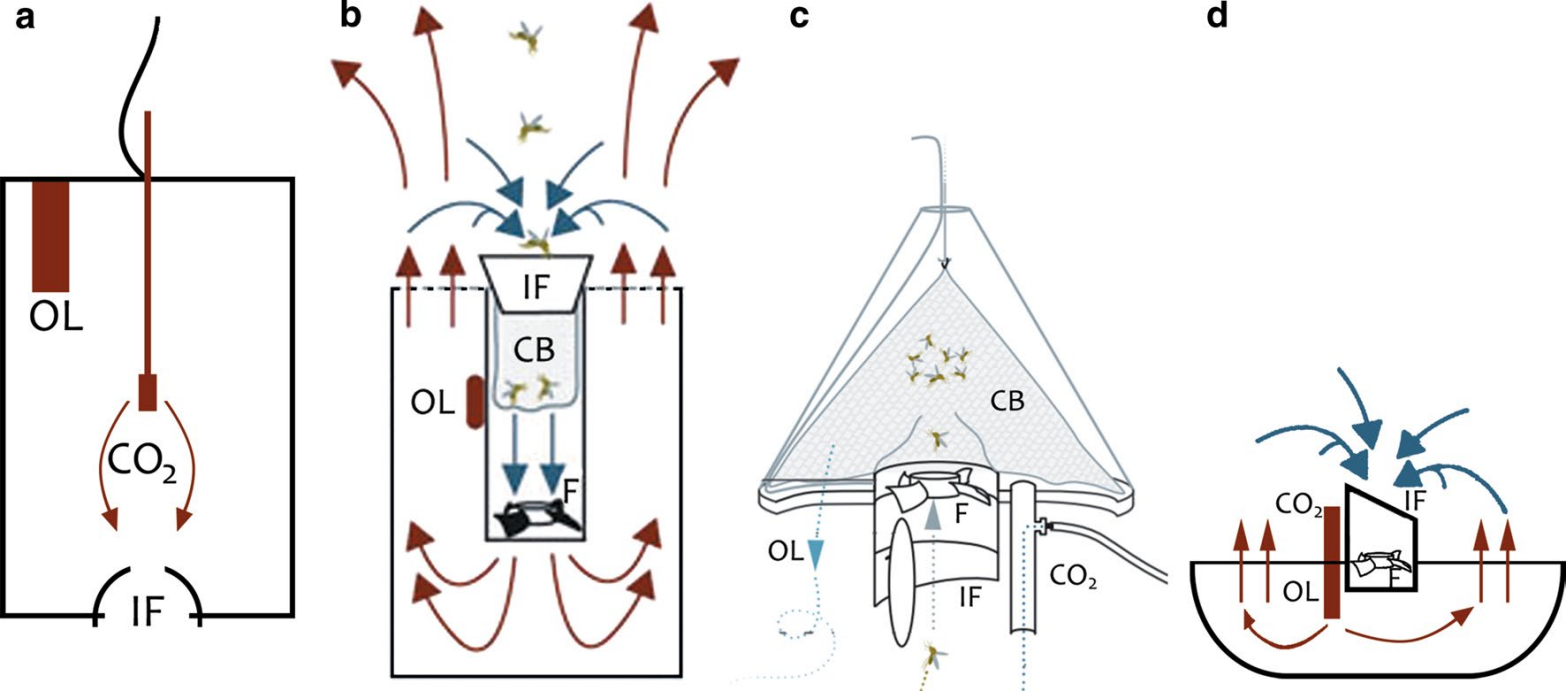
Line drawings of the four mosquito traps used in the experiment. *IF* inlet funnel, *F* fan, *CB* catch bag, *CO*_*2*_ point of CO_2_ release, *OL* odour lure. Arrows indicate air flow. **a** Representation of the Passive Box trap, which releases its odour plume via natural airflow; **b** the BG-Sentinel 2 trap, where mosquitoes are attracted by an odour plume and are sucked into the trap by an electric fan. Mosquitoes are caught in a catch bag and hence remain undamaged by the fan; **c** the BG-Suna trap, which functions essentially the same as the BG-Sentinel 2; however, mosquitoes are sucked upwards, through the fan; **d** the BG-Bowl trap, where air flow patterns and capture mechanism are the same as in the BG-Sentinel 2 trap. Images b, c and d sourced from the manufacturer at (https://eu.biogents.com)

In a follow-up experiment, the catch efficiency of the BG-Sentinel trap, BG-Suna trap and BG-Bowl trap (three fan-powered traps produced by Biogents AG, Germany) were compared to a Passive Box trap [18] (see Fig. 1). The Passive Box Trap was designed to provide mosquitoes with a relatively undisturbed plume of CO_2_ to lure them to the trap, in contrast with fan-powered traps from which the odour plume may be turbulent and more difficult to follow. All traps were equipped with the same lure combination of CO_2_ gas at 250 ml/min and the odour lure which attracted most mosquitoes in the previously described odour lure comparison experiment. The four traps were evaluated and captured mosquitoes processed as described above, i.e., in a 4-by-4 Latin square trial, with 16 repetitions in the same locations as the previous study with traps running between 16.00 and 08.00 h. In addition, the ease with which mosquitoes could be identified was assessed qualitatively.

### Statistical analysis of the data

For both the lure comparison and the trap comparison study, the impact of the different sampling locations and sampling nights on the catch sizes were analysed by a forward-selection Generalized Linear Mixed Model (GLMM) with a Poisson distribution. Associations between odour lure type or trap type and *An. farauti* catch sizes were sequentially analysed by means of a Friedman test for repeated measures, with sampling nights as a random factor to account for fluctuations in mosquito densities, followed by a dedicated post hoc analysis to study individual differences between the experimental treatments. All statistical analyses were conducted in RStudio (R v. 3.4.1).

The effect of weather on catch sizes of *An. farauti* was evaluated using a forward-selection GLMM. The fixed factors were mean nightly temperature, mean humidity per night, amount of rainfall per sampling night, and the rainfall 2 weeks prior to each sampling night. The first three factors were expected to affect mosquito flight activity, whereas the last parameter was assumed to influence the number of larval habitats and hence could affect the number of newly hatched adults available to trap 2 weeks later.

## Results

### Lure comparison study

During this experiment, a total of 10,297 mosquito females were captured. The most common species sampled was *An. farauti* s.l. (n = 3455), followed by species in the genera *Culex* (3111), *Aedes* (2037), *Verrallina* (1233), *Coquilettidia* (345), *Mansonia* (87), other *Anopheles* (23), and *Tripteroides* (6). Trap location did not significantly influence *An. farauti* catch numbers (GLMM, z-value = 1.389, p = 0.08). However, there was a significant association between sampling night and *An. farauti* catch size (GLMM, z-value = 2.355, p < 0.01). Hence, the differences between sampling nights were taken into account in further analyses.

**Fig. 2:**
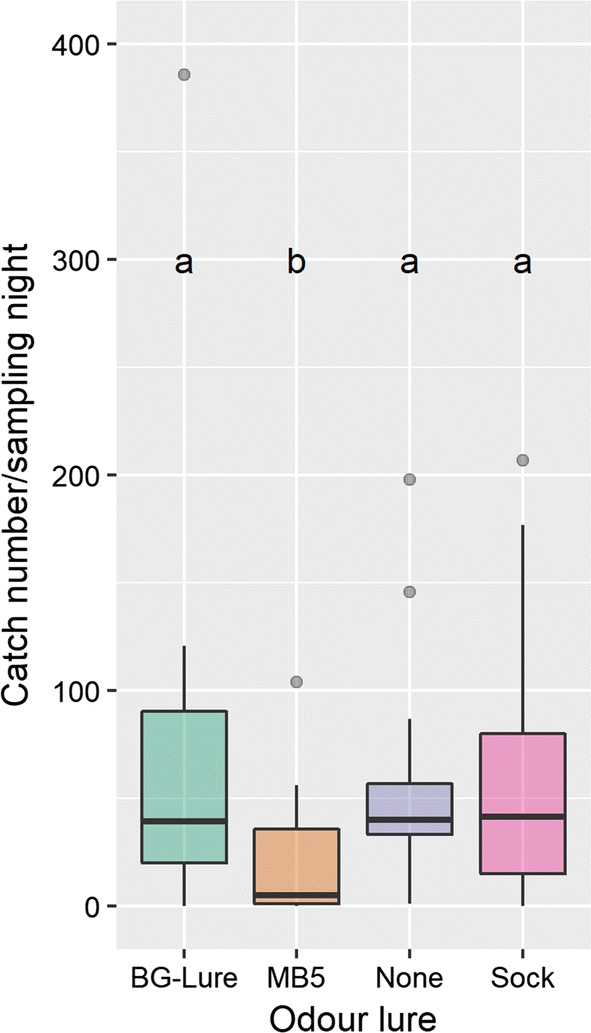
The number of *Anopheles farauti* caught per odour lure type. Catch sizes with the MB5 treatment were 3 times lower than the other treatments over 16 sampling nights. Whiskers indicate spread in number of trapped females per night; letters indicate statistically significant differences. All odour treatments were combined with CO_2_ for maximal effectiveness

The total numbers (and percentages) *of An. farauti* captured in the Suna trap baited with different lures were: 1179 (31.8%) with CO_2_ + BG-Lure, 1040 (31.4%) by the CO_2_ + sock, 344 (9.0%) with CO_2_ + MB5, and 892 (27.5%) with CO_2_ alone (Fig. 2). The Suna trap with CO_2_ + MB5 lure yielded catch sizes which were on average three times lower than the other three traps, including the control (Friedman test, Friedman χ^2^ = 11.35, p = 0.01; post hoc pair-wise comparison, p = 0.043) (see Fig. 2). The addition of a BG-Lure cartridge or a natural host odour did not significantly affect *An. farauti* catch numbers, compared to the control, which used CO_2_ alone. As the trap baited with a BG-Lure cartridge and CO_2_ yielded most mosquitoes, this combination was selected for use to evaluate different trap designs.

### Trap comparison study

**Fig. 3:**
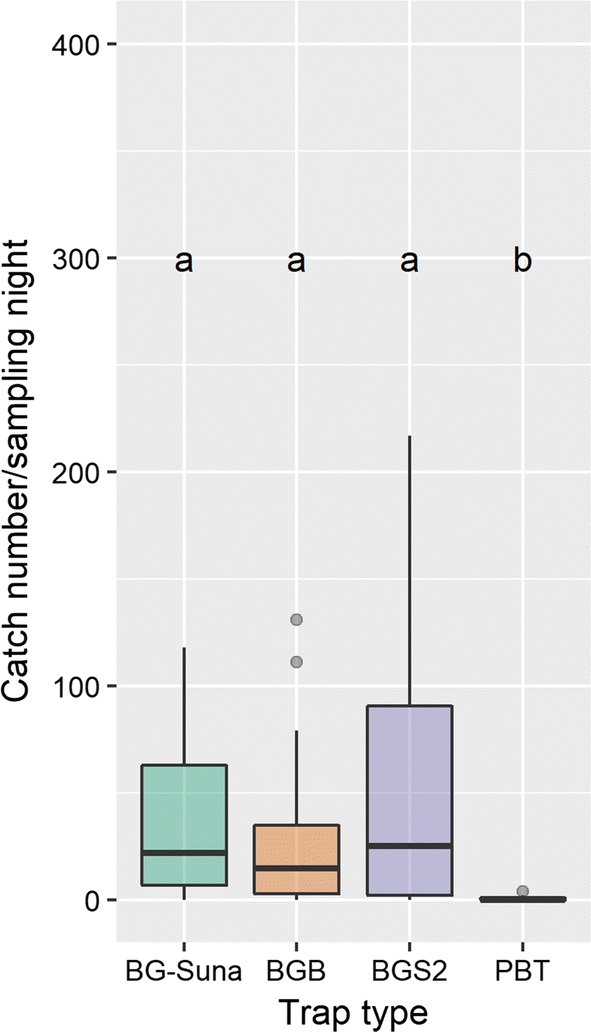
The number of *Anopheles farauti* caught per trap type. *BG-Suna* BG-Suna trap, *BGB* BG-Bowl trap, *BGS2* BG-Sentinel 2 trap, *PBT* Passive Box trap. Catch size is significantly lower for the PBT (Friedman, n = 64, p < 0.001). Whiskers indicate spread in number of trapped females per night; letters indicate statistically significant differences

In the comparative evaluation of the BG-Sentinel, BG-Suna and BG-Bowl traps against a Passive Box Trap, 12,659 mosquito females were caught over 16 nights, of which 1972 were *An. farauti*. The other mosquitoes present in the traps belonged to the genera *Culex* (5406), *Aedes* (2570), *Verrallina* (2449), *Coquilettidia* (213), *Mansonia* (30), *Anopheles* (11), and *Tripteroides* (8). Just under half (n = 838, 42.5%) of the captured *An. farauti* were collected by the BG-Sentinel 2 trap, 620 (31.4%) with the BG-Suna trap, 503 (25.5%) in the BG-Bowl trap and 11 (0.6%) in the Passive Box trap. A significant effect of sampling night on catch number was found (GLMM, z-value = 2.811, p = 0.002), but there were no location effects. The Passive Box trap caught significantly fewer *An. farauti* compared to the other traps (Friedman test, Friedman χ^2^ = 21.178, p < 0.001). Differences in catch size between the different fan-powered traps were not statistically significant (post hoc comparisons p > 0.05) (see Fig. 3).

In general, the condition of trapped mosquitoes enabled identification on the basis of physical characteristics. Mosquitoes captured by the BG-Sentinel 2 trap, BG-Suna trap and Passive Box trap were usually undamaged. However, samples from the BG-Bowl trap were often damaged, with mosquitoes suffering mechanical damage evidenced by severed legs or abdomens. In addition, accumulated moisture inside the trap, which mainly occurred on humid or rainy nights, negatively affected the condition of mosquitoes. In this region, there are clear morphological differences between the local species so most specimens collected using the BG-Bowl trap were still identifiable.

Mosquito sampling took place during the relatively cool and dry months of June and July. Mean night temperatures varied between 18.1 and 23.8 °C, and mean humidity varied between 56.6 and 84.4%, with recorded rainfall on 9 of the 32 sampling nights, varying between 0.2 and 5.4 mm. Rainfall on the sampling night did not affect mosquito catch numbers (GLMM, n = 128, z-value = − 0.599, p = 0.54). However, temperature (GLMM, n = 128, z-value = 3.455, p < 0.001), humidity (GLMM, n = 128, z-value = − 19.107, p < 0.0001) and rainfall 2 weeks prior to sampling (GLMM, n = 128, z-value = 8.883, p < 0.0001) correlated with *An. farauti* catch numbers. Lower catch numbers were associated with colder and more humid nights. In contrast, rainfall which occurred 2 weeks before a sampling night was associated with higher *An. farauti* catch numbers.

## Discussion

*Anopheles farauti* s.l. were captured using three different, commercially available, fan-powered traps baited with CO_2_ and an odour lure. Although all three odour lures attracted female mosquitoes, CO_2_ combined with the BG-Lure or a natural human odour, or CO_2_ alone attracted three times more females than the MB5 lure combined with CO_2_. The MB5 odour lure is a blend, which was developed to attract *Anopheles* females [19]. However, the MB5 blend was designed for and tested on African *Anopheles* species, specifically *Anopheles gambiae* s.s. and *Anopheles coluzzii* [20]. These species are both highly anthropophilic [21], whereas tests on *Anopheles arabiensis*, which shows opportunistic feeding behaviour, resulted in much more variation in catch numbers when the MB5 lure was used [20]. Similar to *An. arabiensis*, *An. farauti* displays opportunistic feeding behaviour [22]. This may explain the lower attractiveness of the MB5 blend in this experiment.

*Anopheles farauti* opportunistic blood feeding may have evolved because potential hosts vary greatly by location and relative abundance, hence females attracted by odorous compounds that are produced by many mammalian blood host species are more likely to be successful in finding a blood meal. These findings show that traps baited with CO_2_ and a natural host odour, CO_2_ and the synthetic BG-Lure or CO_2_ alone all yielded high catch sizes. However, it is unlikely that host-seeking females follow CO_2_ alone when searching for a blood meal.

*Anopheles farauti* is only found on the Australian side of the Wallace line, indicating a very early spatiotemporal separation between this species and its African kin. Therefore, both evolutionary lines may have developed attraction to a different, specific combination of olfactory compounds.

All of the fan-powered traps tested (BG-Sentinel, BG-Suna and BG-Bowl) captured high numbers of *An. farauti* females over the course of the experiment. The traps were all equipped with a BG-Lure cartridge and CO_2_, as this combination yielded the highest catch size in the first experiments. The results suggest that fan-powered trapping of Australasian malaria mosquitoes could have the potential to replace human landing catches, but studies to compare traps with HLCs are required to validate this conclusion.

Catch sizes in the fan-powered traps were significantly higher than numbers captured by the Passive Box trap and it was noted that samples from the BG-Sentinel 2 and BG-Suna trap were in better condition than samples from the BG-Bowl trap. To enable easy and accurate identification in the field, sampled mosquitoes need to remain relatively undamaged. A well-known issue for fan-powered traps is that they can seriously damage mosquitoes during collections [23]. In the BG-Sentinel 2 trap, this is prevented by positioning of the mesh catch bag directly behind the inlet funnel, so mosquitoes are captured before they pass through the fan (see Fig. 1b). Mosquitoes pass through the fan in both the BG-Suna and the BG-Bowl trap (see Fig. 1c, d), making morphological identification more challenging.

The BG-Suna trap is a weather-resistant trap hanging from a rope, whereas the BG-Bowl trap is essentially a simplified version of the BG-Sentinel 2 trap, with a perforated cover which makes these traps less rain-resistant than the BG-Suna trap. In addition, the BG-Bowl trap does not possess a catch bag and mosquitoes are collected in the main body of the trap where water can accumulate and cause morphological damage. Indeed, it was observed that the condition of mosquito samples from the BG-Bowl trap was better after a sampling night without precipitation, whereas this difference was not observed in the BG-Suna trap.

Despite their ready availability and advantages, fan-powered traps are used rarely in the Southwest Pacific region. Limited usage of traps may be a function of trap cost, as well as requirements for a power source (usually a battery that needs regular replacement) and CO_2_. As the results of this study have shown, a source of CO_2_ is an essential component in a trap designed to lure host-seeking mosquitoes in the Southwest Pacific, as has been shown previously for *An. coluzzii* [24]. Traditional sources of CO_2_, such as compressed gas or dry ice, are expensive, cumbersome and not readily available in places such as the Solomon Islands where tools for monitoring malaria vectors are urgently needed. CO_2_ produced by yeast fermentation of sugar or molasses has been an effective solution in Kenya where traps are used to monitor malaria vectors in the field where dry ice and compressed gas are difficult to use [25].

All three of the fan-powered traps tested in this study attracted large numbers of *An. farauti* as well as other mosquito species, including *Aedes vigilax*, *Verrallina carmenti* and *Verrallina funerea*. These three species are important vectors of Australian vector-borne diseases such as Ross River Virus [26]. The high sampling yield of fan-powered traps shows their potential for vector monitoring in the Southwest Pacific. Targeting multiple key vector species using one trap could make future surveillance studies much more efficient, since it enables accurate, simultaneous population monitoring of a range of important disease vectors. Future studies should be conducted to compare capture rates of traps against HLCs for *An. farauti* in the field, in areas with both low and high background densities of *An. farauti*. Comparisons between traps and HLCs would allow confirmation of whether traps could replace traditionally used HLCs.

## Conclusions

The use of fan-powered traps such as the BG-Suna trap or BG-Sentinel 2 trap, baited with CO_2_ and an appropriate odour lure, can be a promising solution to increase vector monitoring in the Southwest Pacific. BG-Suna traps equipped with CO_2_ alone, CO_2_ plus a BG-Lure cartridge, or CO_2_ plus a natural host odour perform equally well in the field with respect to *An. farauti* catch sizes in the field.

## Supplementary information

**Additional file 1.** Complete dataset for lure comparison and trap comparison study.

## Data Availability

All data generated or analysed during this study are included in this published article [and its additional information files] (Additional file 1).

## Abbreviations

BG: Biogents AG
CDC: Centers for Disease Control
CO_2_: carbon dioxide
GLMM: Generalized Linear Mixed Model
HLC: human landing catch
IRS: indoor residual spraying
LLIN: long-lasting insecticidal net
MB5: Mbita 5-component lure
